# Dissatisfaction with Veterinary Services Is Associated with Leopard (*Panthera pardus*) Predation on Domestic Animals

**DOI:** 10.1371/journal.pone.0129221

**Published:** 2015-06-26

**Authors:** Igor Khorozyan, Mahmood Soofi, Amirhossein Khaleghi Hamidi, Arash Ghoddousi, Matthias Waltert

**Affiliations:** 1 Workgroup on Endangered Species, J.F. Blumenbach Institute of Zoology and Anthropology, Georg-August University of Göttingen, Göttingen, Germany; 2 Persian Wildlife Heritage Foundation, Tehran, Iran; Institut Pluridisciplinaire Hubert Curien, FRANCE

## Abstract

Human-carnivore conflicts challenge biodiversity conservation and local livelihoods, but the role of diseases of domestic animals in their predation by carnivores is poorly understood. We conducted a human-leopard (*Panthera pardus*) conflict study throughout all 34 villages around Golestan National Park, Iran in order to find the most important conflict determinants and to use them in predicting the probabilities of conflict and killing of cattle, sheep and goats, and dogs. We found that the more villagers were dissatisfied with veterinary services, the more likely they were to lose livestock and dogs to leopard predation. Dissatisfaction occurred when vaccination crews failed to visit villages at all or, in most cases, arrived too late to prevent diseases from spreading. We suggest that increased morbidity of livestock makes them particularly vulnerable to leopard attacks. Moreover, conflicts and dog killing were higher in villages located closer to the boundaries of the protected area than in distant villages. Therefore, we appeal for improved enforcement and coordination of veterinary services in our study area, and propose several priority research topics such as veterinarian studies, role of wild prey in diseases of domestic animals, and further analysis of potential conflict predictors.

## Introduction

Human-wildlife conflicts over livestock predation, crop raiding or attacks on humans are relatively common and well documented [1]. Financial and social losses from conflicts can be significant, especially for poor communities with meager opportunities for alternative incomes, thus provoking hostile attitudes and persecution of culprit animals [2]. On the other hand, the species of concern include officially protected flagship and umbrella species (e.g., African elephant *Loxodonta africana*, large felids of *Panthera* genus), keystone species disliked by some sectors of the public (e.g., gray wolf *Canis lupus*) or lower-level biodiversity (e.g., monkeys, birds and ungulates). In this case, conflicts challenge a balance between nature conservation and livelihoods and require tremendous long-term efforts to maintain or restore the integrity of the environment in socially acceptable ways [3].

Human-carnivore conflicts over predation on domestic animals are particularly well-known. Such conflicts depend on many factors such as densities of carnivores, wild prey and livestock, habitat and livestock characteristics, socio-economic conditions and husbandry practices [1], [4–5]. Surprisingly little empirical knowledge exists about the relationships between predation and diseases of domestic prey though diseases are among the major problems for animal husbandry throughout the world, especially in developing countries [6–11]. While theoretical modeling of predator-prey-disease relationships is well-developed and consistent, the limited empirical studies demonstrate contradictory results that predators can either keep diseases down (“healthy herds” hypothesis) or do the opposite by building up the disease burden of prey hosts [12–14].

Predicting human-carnivore conflicts is an important practical task, though its development began only recently and diseases are not yet addressed as potential predictors [8], [15–17]. In the context of diseases and conflicts, the availability of true absence data (i.e., no conflicts) is of utmost importance. It is easier to obtain absence data for conflicts than, for example, for a species especially if it is rare or mobile [18]. For this, it is just required to conduct sweeping surveys over all local settlements and to record where conflicts exist and where they do not. In this case, the application of presence-only modeling approaches such as maximum entropy (MaxEnt) or ecological niche factor analysis (ENFA) is not justified and the presence-absence analysis should be used instead [18]. Moreover, presence-only models are prone to errors when their pseudo-absence records are inaccurate, i.e. they may contain true presence data, and presence data are spatially biased [19]. When all local settlements are surveyed for conflicts, a presence-absence study transforms into a case-control study, which produces an absolute and most reliable probability of conflict in a given area [20].

In this paper, we provide the results of a case-control study of human-leopard (*Panthera pardus*) conflict in Iran. We describe the conflict pattern, estimate the effect of veterinary services and other variables on the conflict through predictive modeling, and discuss the practical aspects of the model output. Ultimately, we consider conservation implications of this study and propose practical actions to mitigate the conflict.

## Materials and Methods

### Study Area

**Fig 1:**
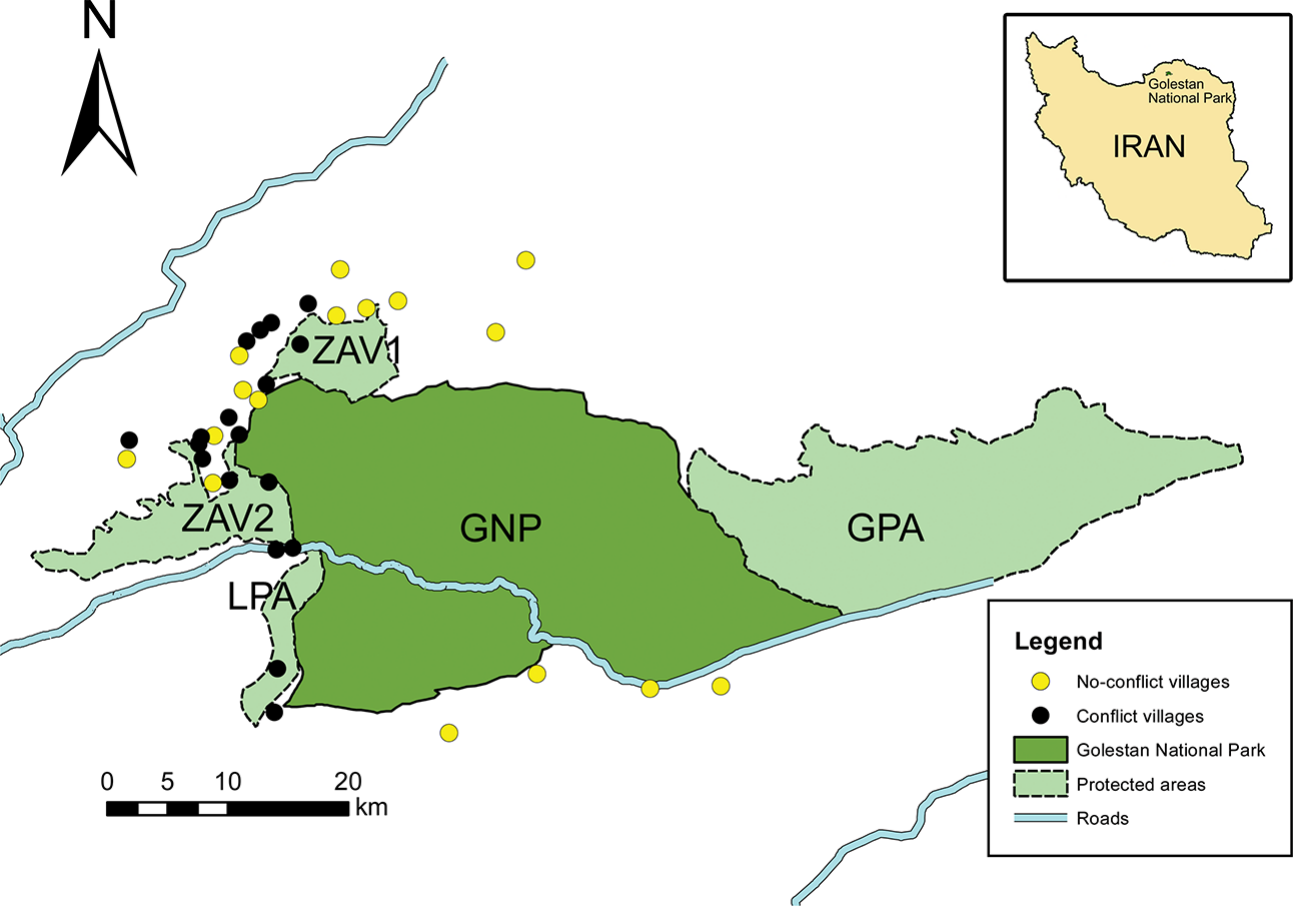
Location of Golestan National Park (GNP), Ghorkhod Protected Area (GPA), Zav 1 Protected Area (ZAV1), Zav 2 Protected Area (ZAV2), Loveh Protected Area (LPA) and villages with or without human-leopard (*Panthera pardus*) conflict.

This study was conducted in 34 villages located in the Madarsou (Dough) River watershed around Golestan National Park (GNP, 874.02 km^2^) in north-eastern Iran (Fig 1). GNP abuts on Ghorkhod Protected Area (GPA, 431.50 km^2^) in the east, Zav 1 Protected Area (ZAV1, 50.08 km^2^) in the north-west, Zav 2 Protected Area (ZAV2, 93.15 km^2^) in the west and Loveh Protected Area (LPA, 35.89 km^2^) in the south-west (Fig 1). From west to east, elevations increase from 450 m to 2411 m above sea level and precipitation decreases from 700 mm to 150 mm. The main landscape zones are lush humid temperate Hyrcanian forest in the west, steppe in the central part and semi-desert in the east, with the mean annual air temperature 11.5–17.5°C [21]. Local people belong to Turkmen and Persian ethnic groups. They raise cattle, sheep and goats and keep dogs to guard livestock. Dogs are not specifically trained to protect livestock and local culture prohibits buying or selling dogs, including well-trained individuals of appropriate breeds. Shepherds usually take larger dogs to accompany their stock, which, however, do not guarantee good protection. Human population, livestock numbers and holdings, and other characteristics of GNP villages are provided in S1 Table. High-quality stud cattle graze inside villages, whereas other cattle and all sheep and goats move out into mountains and return to stay at night in pens. In June-July, cattle graze near villages on wheat stubble fields and stay away from gadflies swarming in dense vegetation, but later return to mountains after fields are tramped down (in most places) or converted into rice paddies (some lowlands).

### Ethics Statement

This project was reviewed and approved by the boards of Persian Wildlife Heritage Foundation, Iranian Department of Environment and Golestan National Park, in terms of project design and communication with respondents. No written permits were required for the project as it was implemented on unprotected community lands. All people whom we asked to participate gave their verbal consent, therefore filling up a questionnaire form for a respondent meant his consent. No written consent was obtained in an attempt to establish good unofficial relationships with culturally sensitive local people, which was essential to ensure study feasibility. This consent procedure was approved by the above-listed organizations. The respondents were informed beforehand about the purpose of questionnaire surveys, anonymity and security of their information and that this study was unrelated to governmental programs such as compensation or environmental compliance schemes. Interviews were conducted in full compliance with local traditions and ethical requirements, with a local scientist MS being fully involved as a Turkmen/Persian/English translator. No animal handling was conducted.

### Data Collection

Structured questionnaire surveys were conducted in March and May 2013 among 41 council members of 34 villages, all of which are situated outside of GNP on unprotected lands. In our sample, they were 41.4 ± standard error (SE) 1.4 year-old men (range 25–74). These 41 persons represented the joint and agreed opinions of three council members (*Showra*) and one village head (*Deh-yar*) from each of 34 surveyed villages, so our survey practically covered 136 persons. Council members and village heads are the most respected, influential and knowledgeable persons elected by locals to represent their villages. Information provided by them was retrieved from the village databases.

The response variables included conflict, killing and predation rates in each village. Conflict was a categorical variable (1, yes and 0, no) defined as a situation in which at least one individual of cattle, sheep, goats and/or dogs was killed by leopards in a village during one year (March 2012 to March 2013). Killing also was categorical (1, yes and 0, no) and defined like conflict, but measured separately for cattle, sheep and goats, and dogs. Cattle, sheep and goats, and dog predation rates indicated the numbers of these animals killed by leopards in a village during the same year. We also studied the relationships between the response variables and poaching of large mammals, including leopards. Poaching was categorized as present (1) or absent (0) in a given village based on data provided by informants, not by the interviewed council members.

The predictor variables included the following characteristics for each village: satisfaction with veterinary services (1, yes and 0, no), vegetation cover (1, overgrown by 0–25% and 0, overgrown by >25%), decimal latitude, decimal longitude, elevation (m), distance to protected areas (km), accessibility (km), pasture size (ha), number of livestock owners (persons), percentage of livestock owners to all villagers, number of shepherds (persons), percentage of shepherds to villagers, number of cattle (individuals), number of sheep and goats (individuals), number of dogs (individuals), number of households (units), population (persons), number of men (persons) and percentage of men to all villagers (S1 Table).

Distance to protected areas was taken in order to test whether conflict, killing and predation rates are stronger near the boundaries of protected areas. We measured this distance from each village to GNP, GPA, ZAV1, ZAV2 and LPA, whichever is the closest, in ArcGIS 10.1 on the digitized map of GNP. This map was created from SRTM 90 m Digital Elevation Model (DEM) and 300 m land cover map of the CGIAR Consortium for Spatial Information (http://srtm.csi.cgiar.org) and then resampled at 250 m. Persian Wildlife Heritage Foundation provided the GIS files of protected area boundaries. Village accessibility was measured in two ways, depending on village location. For 31 villages situated in the north-west, north, south-west and partly south of GNP, accessibility was a road distance from a village to Tangerah, the village from where all local roads divert. Accessibility of three other villages in the south-east was measured as a distance to the highway (Fig 1). Tangerah is the only local village having its veterinarian facility, but due to its limited capacity veterinarian services are provided from the neighboring towns of Kalaleh and Galikesh. The number of village households and demographic data were obtained from the Statistical Centre of Iran (http://www.amar.org.ir).

### Data Analysis

Chi-square (χ^2^) test and Mann-Whitney test were used to estimate differences in categorical and continuous variables, respectively, between conflict and no-conflict villages. The odds ratio was calculated to assess the magnitude of the effects of the variables in presence (conflict villages) vs. absence (no-conflict villages) samples [7, 20]. For example, the odds ratio of 0.10 describing the effect of satisfaction with veterinary services on conflict means that this satisfaction reduces the probability of conflict by 0.90 or 90%.

Correlation between variables was estimated by Spearman’s rank correlation coefficient (r_s_) and uncorrelated biologically more meaningful variables were analyzed by Generalized Linear Models (GLM). The GLMs were applied to describe the relationships between predictors and response variables, using the Poisson model for count data of continuous response variables (predation rates) and the logistic regression model for binary data of categorical response variables, viz. conflict and killing [22]. Villages with Cook’s distance (D_cook_) > 1 were considered as outliers and excluded from further re-analysis [22]. The area under Receiver Operating Characteristic (AUC), percentage of correct classification and the likelihood-ratio test were applied to validate the predictive power of the GLMs. A score AUC = 0.5 means that the model has no discriminatory ability and AUC = 1 means that presences and absences are perfectly discriminated [17].

Statistical analysis was done in SPSS 17.0 (IBM, Armonk NY, USA) using two-tailed significance level *P*.

## Results

Out of 34 villages, 18 villages had conflicts with leopards and 16 did not (Fig 1). In conflict villages, local people correctly assigned livestock and dog kills to leopards from personal observations of leopards on carcasses (13 villages, 72.2%), throat bite marks on kills (11, 61.1%), signs of disemboweling (3, 16.7%), prey concealed on tree (3, 16.7%), leopard sounds (2, 11.1%), leopard tracks (2, 11.1%) and claw marks on victim’s neck (1, 5.6%). In most cases (17 villages, 94.4%), livestock and dogs were killed in the mountains far from their villages.

In total, 24 calves, 119 sheep and goats, and 30 dogs were killed by leopards in 18 conflict villages in 2012–2013. Forty-one cases of leopard predation (1–5 cases/village) were recalled and described with sufficient details. Presence of shepherds and dogs did not eliminate leopard attacks, since shepherds were present in 23 of them (56.1%) and absent in 18 (43.9%); dogs were present in 21 cases (51.2%) and absent in 20 (48.8%). When shepherds were present during leopard attacks, they reacted by shouting with or without throwing stones in 21 cases (91.3%) and stayed unaware of leopards in two cases. When dogs were present, they ran away with or without barking (10, 47.6%), were killed without resistance (4, 19.0%), barked from afar (3, 14.3%), approached but then retreated (3, 14.3%) or did not react (1, 4.8%).

**Fig 2:**
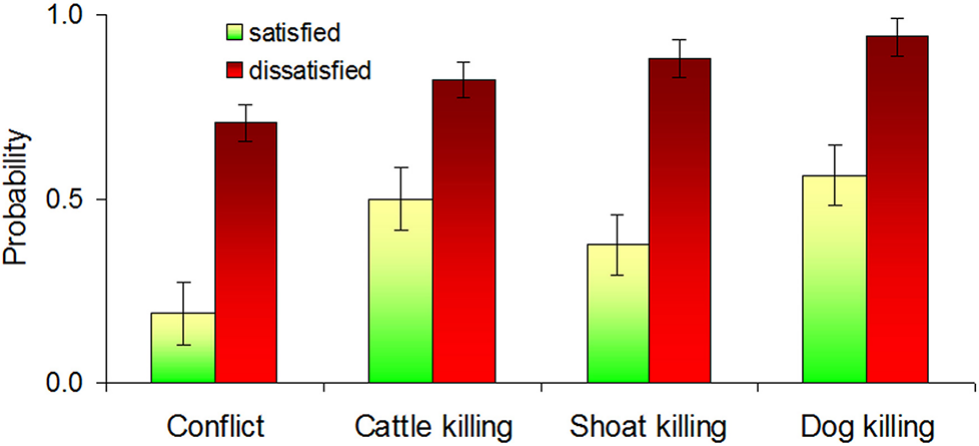
The effect of satisfaction and dissatisfaction with veterinary services on the probabilities of conflict, cattle killing, sheep and goat (shoat) killing and dog killing modeled by logistic regression. The error bars indicate standard error.

Satisfaction with veterinary services was the only predictor making a significant effect on conflict and killing, but not on predation rates, which were not affected by predictors we used. Conflicts, cattle killing, sheep and goat killing, and dog killing were higher in villages where respondents were dissatisfied with veterinary services, but this relationship was less evident in cattle (Table 1; Fig 2). Logistic regression confirmed that predictability of cattle killing by leopards was lower than predictability of conflict or killing of sheep, goats and dogs (Table 2). Satisfaction with veterinary services was not correlated with accessibility of villages (r_s_ = 0.099, *P* = 0.585). There was no relationship between poaching and conflict (χ^2^ = 2.892, *P* = 0.168), satisfaction with veterinarian services (χ^2^ = 0.036, *P* = 1.000), cattle killing (χ^2^ = 2.555, *P* = 0.152), sheep and goat killing (χ^2^ = 0.216, *P* = 0.729) and dog killing (χ^2^ = 0.384, *P* = 0.693).

**Table 1:** The significant effects of satisfaction with veterinary services on human-leopard (*Panthera pardus*) conflict, cattle killing, sheep and goat killing, and dog killing by leopards in villages around Golestan National Park, Iran.

Response variable	Chi-square χ^2^	Significance level *P*	Odds ratio
**Conflict**	8.93	0.003	0.10
**Cattle killing**	3.88	0.049	0.21
**Sheep and goat killing**	9.17	0.002	0.08
**Dog killing**	6.44	0.011	0.08

**Table 2:** The models predicting the probabilities of human-leopard conflict and killing of cattle, sheep and goats, and dogs by leopards from the villagers’ satisfaction with veterinary services.

Response variable	Equation	Parameter estimates	%	AUC	Likelihood-ratio test
**Conflict**	p = 1/(1 + exp(2.342×VS– 0.875))	Wald = 7.907, *P* = 0.005	75.8	0.76	χ^2^ = 9.435, *P* = 0.002
**Cattle killing**	p = 1/(1 + exp(1.540×VS– 1.540))	Wald = 3.624, *P* = 0.057	66.7	0.68	χ^2^ = 3.985, *P* = 0.046
**Sheep and goat killing**	p = 1/(1 + exp(2.526×VS– 2.015))	Wald = 7.655, *P* = 0.006	75.8	0.77	χ^2^ = 9.777, *P* = 0.002
**Dog killing**	p = 1/(1 + exp(2.521×VS– 2.773))	Wald = 4.829, *P* = 0.028	75.8	0.76	χ^2^ = 7.018, *P* = 0.008

Abbreviations: AUC–area under the Receiver Operating Characteristic (ROC), p–probability of response variable, *P*–significance level, VS–satisfaction with veterinary services (1 if satisfied, 0 if not), %–percentage of correct classification.

Out of 16 villages, which were dissatisfied with veterinary services, state vaccination crews did not visit five villages (31.3%) and came too late in 11 others (68.7%). As the interviewed council members claimed, in both these cases livestock became affected by diseases, which would be otherwise prevented by timely vaccination or treatment. Alternative private veterinarians are expensive and not affordable to villagers. Dogs are not vaccinated in GNP villages.

Conflicts were higher (Mann-Whitney = 79.000, Z = -2.243, *P* = 0.025) and satisfaction with veterinary services was lower (r_s_ = 0.382, *P* = 0.028) in villages with lower longitudes, i.e. in the west of GNP. Conflict and dog killing were inversely correlated with distance to protected areas (r_s_ = -0.382, *P* = 0.010 and r_s_ = -0.435, *P* = 0.010). Conflict, killing and predation rates of livestock and dogs were not correlated with vegetation cover (r_s_ from -0.304 to 0.075, *P* from 0.081 to 0.789).

## Discussion

This study is the first empirical evidence demonstrating that villages in which people are dissatisfied with veterinary services are more likely to lose livestock and dogs to leopard attacks (Fig 2; Tables 1 and 2). Hostility towards big cats may inflate their perceived culpability by assigning other mortality causes to predation, but in our study the description of leopard predation signs was apparently accurate. Predictability of cattle killing was lower than that of small livestock and dogs, possibly because of higher tolerance of cattle to diseases in humid conditions of villages to the west of GNP where most conflicts occurred (Fig 1). Notably, it was possible to predict probabilities of conflict or killing, but not absolute numbers of killed livestock and dogs.

In Iran, veterinary services are provided by state-supported vaccination crews. These crews are authorized to visit villages at least once a year for anthrax and foot-and-mouth vaccination and for disease diagnostics and treatment, e.g. administration of drugs against worms such as *Fasciola hepatica* and *F*. *gigantica*. Fascioliasis and foot-and-mouth disease are common throughout Iran, being among the highest concerns for national agriculture [23–25]. Dissatisfaction with these services was common in our study, but not confined to hardly accessible remote villages, as only few of them were not visited at all. In most cases, villagers complained that vaccination crews come too late when diseases are already spread and belated vaccination is ineffective. Services provided by private veterinarians are mostly unaffordable. We suggest that poor veterinary treatment contributed to increased morbidity of livestock, which, in its turn, made them increasingly vulnerable to leopard attacks. The most common disease mentioned by respondents was hoof infection caused by *Fusobacterium necrophorum*, which was told to impede free movements of livestock. This disease was widespread in humid forest landscapes of villages to the west of GNP. Perhaps, hoof infection also limited the fleeing behavior of livestock and exposed them to higher predation. Fascioliasis, echinococcosis and tick-borne diseases are also widespread in Golestan Province where all conflict villages near GNP are located [24], [26–29].

Albeit we demonstrated higher conflict and killing of domestic animals in disease-affected villages, it is still unclear whether leopards select diseased individuals from the stock or take individuals randomly in a diseased stock. It is imperative to investigate the occurrence of diseases in living domestic animals vs. leopard kills and their impact on leopards and their relationships with key competitors (wolves) and wild prey. We are aware of only one work describing parasite infestation, but not diseases or their consequences, in leopards of GNP [30].

A particular concern is the fidelity of leopards to taking dogs in affected villages. Because dogs have not been vaccinated in our study area, high level of their off-take in villages with unsatisfactory veterinary services could be caused by leopards, which are attracted by morbid livestock. Dogs can act as reservoirs of diseases to which leopards are vulnerable and preying on them provides ideal circumstances for disease transmission [31]. Although leopards are known to be low-affected by diseases because of their strict solitary life and rarity, which limit mutual contacts, they can still be infected from dogs by canine distemper, rabies and echinococcosis [32–36]. However, solitude is not a salvation as now canine distemper threatens another solitary felid, the Siberian tiger (*P*. *tigris altaica*), and thus may impinge on co-existing Amur leopard (*P*.*p*. *orientalis*) [37]. Additionally, leopards can develop tuberculosis and mange or become infested by *Echinococcus* spp. by consuming diseased livestock [34]. A tick-borne protozoan *Babesia*, which is common in Golestan Province, can trigger mortality from canine distemper as found in African lions (*P*. *leo*) [27, 38]. Disease transmission can spread fast due to the habit of carnivores to attack domestic animals repeatedly from the same households [15].

This study is an appeal to improve the effectiveness of veterinary services so as to minimize livestock and dog losses to diseases and leopard predation. Particular attention should be paid to dog vaccination. As local villages are situated in three provinces (Golestan, North Khorasan and Semnan), good coordination between their administrative bodies is required to ensure timely vaccination, diagnostics and treatment of domestic animals. Provision of veterinary services to local communities is expected to be an important tool for increasing conservation effectiveness. An earlier study has shown that the improvement of local healthcare infrastructure and services can be a strong boost for conservation [39], and we hope for the same effect also from veterinarian services around GNP.

Quite counterintuitively, we did not find support for the hypothesis that husbandry practices such as herding and the use of guarding dogs might be important predictors of leopard predation on domestic animals in this study. Although the degree of effectiveness of husbandry methods has not been measured, they need to be improved. Dogs are physically not suitable, nor properly trained to withstand leopard attacks around GNP, but local shepherds are not allowed to carry firearms and their attempts to scare leopards away are futile. Permitting the usage of firearms by shepherds is not a solution since it will stimulate poaching and the prescription for preventive air-shooting is hard to control. Our results show that conflicts, satisfaction with veterinarian services and poaching are not correlated in GNP, perhaps because poachers target big ungulate trophies, while leopards are shot during chance encounters [40].

We found support for a local belief that proximity of villages to protected areas is a reliable predictor of human-leopard conflicts. Conflicts and dog killing were much stronger around villages which are located closer to protected areas, indicating that leopard predation is higher along their boundaries. However, a belief that presence of overgrown scrubland pastures leads to more conflicts was refuted. More fine-scale research is required to determine the possible effect of other potential conflict predictors which were not measured in this study, such as distances between livestock kill sites and villages, numbers of active shepherd dogs that accompany livestock, and location of kill sites in relation to landscape characteristics and livestock movement routes. Moreover, we feel that differences in dietary preferences and hunting tactics of individual leopards might also be important in local predation patterns.

## Supporting Information

S1 Protocol The questionnaire form. (PDF)

S1 Table Quantitative information on predictor and response variables in 34 villages around Golestan National Park, Iran. (XLS)
