# Correction: Dissatisfaction with Veterinary Services Is Associated with Leopard (*Panthera pardus*) Predation on Domestic Animals

**DOI:** 10.1371/journal.pone.0134886

**Published:** 2015-07-31

**Authors:** The PLOS ONE Staff

There are errors in the Funding section. The correct funding information is as follows: This study was supported by the Mohamed bin Zayed Species Conservation Fund (IK, MS, AKH, AG, http://www.speciesconservation.org/, project No. 12255025) and Erasmus Mundus/ALRAKIS (IK, http://www.alrakis.eu/, projects No. 2011-2577 and No. 2012-2733). Currently, IK and MW receive funding from Alexander von Humboldt Foundation (http://www.humboldt-foundation.de/, project No. 1151598), MS from Erasmus Mundus/SALAM (http://portal.uw.edu.pl/en/web/salam/, project No. 2013-2437-001-001-EMA2), and AG from German Academic Exchange Service (DAAD,https://www.daad.de, project No. A/11/96604) and Panthera Kaplan Graduate Awards (http://www.panthera.org/). Additionally, IK, MS, AKH and AG receive support from Prince Bernhard Nature Fund and Mohamed bin Zayed Species Conservation Fund (project No. 14059333). This publication was supported financially by the Open Access Grant Program of the German Research Foundation (DFG) and the Open Access Publication Fund of the University of Göttingen. The funders had no role in study design, data collection and analysis, decision to publish, or preparation of the manuscript.

The publisher apologizes for the errors.

## References

[1] Khorozyan I, Soofi M, Khaleghi Hamidi A, Ghoddousi A, Waltert M (2015) Dissatisfaction with Veterinary Services Is Associated with Leopard (*Panthera pardus*) Predation on Domestic Animals. PLoS ONE 10(6): e0129221 doi: 10.1371/journal.pone.0129221 26114626 10.1371/journal.pone.0129221 PMC4483275

