# Supplementary material for: Dissatisfaction with Veterinary Services Is Associated with Leopard (Panthera pardus) Predation on Domestic Animals
Source: PLoS One. 2015 Jun 26;10(6):e0129221. doi: 10.1371/journal.pone.0129221 (PMC4483275; doi:10.1371/journal.pone.0129221)
Supplement: S1 Protocol — (PDF) [file pone.0129221.s001.pdf]

QUESTIONNAIRE FORM

Interview #		Date		Coordinates, N			
Village		Elevation, m		Coordinates, E			
No. people present	T:	M:	F:	Ages:			

Size of pastures, ha		Pastures leased (L) or owned (O)?		No. years the pastures are used	
No. livestock owners		No. shepherds		No. cattle	
No. sheep		No. goats		No. donkeys	
				No. horses	

Closest farm-forest distance, m		Closest farm-ravine distance, m		Closest farm-water distance, m	

Coordinates of watering point	N	E	Are pastures overgrown?	No (0%)<br>Slightly (0-25%)<br>Moderately (25-50%)<br>Strongly (>50%)

Is leopard a big problem for your village?	Yes<br>No<br>Partly	In which season is the damage the highest?	Spring<br>Summer<br>Autumn<br>Winter	In which month(s) livestock predation is most intense?	

How do you know that just leopard is responsible for predation?	

Where are most livestock killed? Sort out as 1, 2, 3….

- Village, grazing
- Village, in enclosures
- Freely grazing
- Watering points
- Other areas ________________

Which livestock are mostly killed or injured?

Sort out as 1, 2, 3…

- Cattle
- Calves
- Sheep
- Goats
- Horses
- Foals
- Donkeys
- Colts

Which factors bring most financial losses?

- [ ] Diseases
- [ ] Leopard
- [ ] Wolf / Wild boar / Other species ________________ ________________
- [ ] Other _________ _________

Livestock predation records in 2008-2013

Which are the trends in livestock predation in the past 5 years?

- Increase
- Decrease
- Same

Livestock predation by leopard in 2012-2013:

How many livestock were **killed**?

Cattle		Sheep		Horses		Donkeys	
Calves		Goats		Foals		Colts	

How many livestock were **injured**?

Cattle		Sheep		Horses		Donkeys	
Calves		Goats		Foals		Colts	

Is this loss?	Less than usual<br>About average<br>More than average	This loss for your **village** is	Very big<br>Big<br>Medium<br>Small<br>None	This loss for your **households** is	Very big<br>Big<br>Medium<br>Small<br>None

What happens to killed animals?		What happens to injured animals?	

Five most recent cases of leopard attacks on livestock:

CASE 1, the latest:

Date		Time	Dawn<br>Dusk<br>Daytime<br>Night<br>Approx. time<br>———————	Location	
				Coordinates	N | E
				Elevation, m	

Weather	Clear<br>Cloudy<br>Mist/fog<br>Rain<br>Snow<br>Other	No. and type of livestock attacked – killed or injured?		Dog present	Yes	No
				Man present	Yes	No

Dog behaviour toward predator	No reaction<br>Run away<br>Bark<br>Chase<br>Bite/contact<br>Other (specify)	Human behaviour toward predator	No reaction<br>Shout<br>Chase<br>Shoot<br>Other (specify)

CASE 2:

Date		Time	Dawn<br>Dusk<br>Daytime<br>Night<br>Approx. time<br>____________	Location	
				Coordinates	N / E
				Elevation, m	

Weather	Clear<br>Cloudy<br>Mist/fog<br>Rain<br>Snow<br>Other	No. and type of livestock attacked – killed or injured?		Dog present	Yes	No
				Man present	Yes	No

Dog behaviour toward predator	No reaction<br>Run away<br>Bark<br>Chase<br>Bite/contact<br>Other (specify)	Human behaviour toward predator	No reaction<br>Shout<br>Chase<br>Shoot<br>Other (specify)

CASE 3:

Date		Time	Dawn<br>Dusk<br>Daytime<br>Night<br>Approx. time<br>____________	Location	
				Coordinates	N / E
				Elevation, m	

Weather	Clear<br>Cloudy<br>Mist/fog<br>Rain<br>Snow<br>Other	No. and type of livestock attacked – killed or injured?		Dog present	Yes	No
				Man present	Yes	No

Dog behaviour toward predator	No reaction<br>Run away<br>Bark<br>Chase<br>Bite/contact<br>Other (specify)	Human behaviour toward predator	No reaction<br>Shout<br>Chase<br>Shoot<br>Other (specify)

CASE 4:

Date		Time	Dawn<br>Dusk<br>Daytime<br>Night<br>Approx. time<br>___________	Location	
				Coordinates	N | E
				Elevation, m	
Weather	Clear<br>Cloudy<br>Mist/fog<br>Rain<br>Snow<br>Other	No. and type of livestock attacked – killed or injured?		Dog present	Yes | No
				Man present	Yes | No
Dog behaviour toward predator	No reaction<br>Run away<br>Bark<br>Chase<br>Bite/contact<br>Other (specify)			Human behaviour toward predator	No reaction<br>Shout<br>Chase<br>Shoot<br>Other (specify)

CASE 5:

Date		Time	Dawn<br>Dusk<br>Daytime<br>Night<br>Approx. time<br>___________	Location	
				Coordinates	N | E
				Elevation, m	
Weather	Clear<br>Cloudy<br>Mist/fog<br>Rain<br>Snow<br>Other	No. and type of livestock attacked – killed or injured?		Dog present	Yes | No
				Man present	Yes | No
Dog behaviour toward predator	No reaction<br>Run away<br>Bark<br>Chase<br>Bite/contact<br>Other (specify)			Human behaviour toward predator	No reaction<br>Shout<br>Chase<br>Shoot<br>Other (specify)

How many shepherd dogs are in the village?	Adults (> 1 yr), total		Adults used in livestock guarding (LG)		Pups (≤ 1 yr), total		Pups intended for LG	

Are these dogs good in LG?	Yes<br>No, why?<br>Partly, why?	Breeds	

Where do these dogs come from?		How are they trained?	

What measures are used in this village to reduce predation?<br><br>Sort out as 1, 2, 3…		Dogs
		Shooting animal air
		Patrolling
		Sleeping with livestock enclosures pastures
		Avoiding risky places, which? ____________________
		Removing dead livestock died naturally killed by predators
		Scare devices, which? ______________________
		Putting animals in enclosures at night birth period grazing in fences

Are these measures effective?	Yes<br>No, why?<br>Partly, why?<br><br>_________	What do you suggest to reduce predation?		Do you need assistance in livestock predation issues?	Yes<br>No

What kind of assistance do you need?	

Which are the most problematic diseases for livestock?	

Which vet treatments are available?	Sheep dip<br>Vaccination<br>Medicines<br>Other<br>_________

Are you satisfied with your vet services?	Yes<br><br>No, why? _____________<br><br>Partly, why?___________	How high is financial burden for vet services?	**Village**:<br><br>Very high<br>High<br>Medium<br>Low	**Households**:<br><br>Very high<br>High<br>Medium<br>Low

How good is reporting to provincial DoE about cases of leopard predation?	All cases are reported (100%)<br>More than half of cases are reported (50-100%)<br>Less than half of cases are reported (0-50%)<br>Cases are not reported (0%)

If not all cases are reported, why?	

Is response from DoE?	Fast, 1-3 days<br>Rather slow, 3-10 days<br>Very slow, > 10 days	Action by DoE	
